# 2,10-Bis(3-bromo­phen­yl)-3,7,11,15-tetra­oxa-8,16-diaza­tricyclo­[12.2.1.1^6,9^]octa­deca-1(16),6(18),8,14(17)-tetra­ene

**DOI:** 10.1107/S1600536810028813

**Published:** 2010-07-24

**Authors:** Kwang Ha, Sae Byul Park, Young Ju Lee, Hyung Jin Kim

**Affiliations:** a School of Applied Chemical Engineering, Chonnam National University, Gwangju 500-757, Republic of Korea; b Gwangju Branch, Korea Basic Science Institute, Gwangju 500-757, Republic of Korea

## Abstract

The title compound, C_24_H_20_Br_2_N_2_O_4_, is an 18-membered tricycle including two isoxazole rings. The asymmetric unit contains one half of the formula unit; a centre of inversion is located at the centroid of the compound. The dihedral angle between adjacent isoxazole and benzene rings is 84.0 (2)°. The compound displays intra- and inter­molecular π–π stacking inter­actions between the isoxazole rings, the shortest centroid–centroid distances being 3.837 (3) and 3.634 (3) Å, respectively. The mol­ecules are stacked in columns along the *a* axis with short Br⋯Br contacts [3.508 (1) Å].

## Related literature

For the biological activity of isoxazole derivatives, see: Kim *et al.* (1994 ▶, 1997 ▶); Lang & Lin (1984 ▶). For the syntheses of various pyrano[3,4-*c*]isoxzole derivatives, see: Kim *et al.* (1999 ▶).
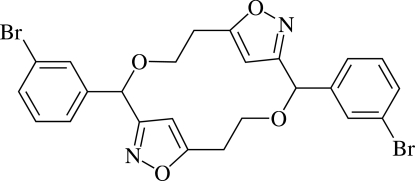


## Experimental

### 

#### Crystal data

C_24_H_20_Br_2_N_2_O_4_
*M* *_r_* = 560.24
Triclinic, 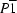

*a* = 5.6446 (4) Å
*b* = 7.3703 (5) Å
*c* = 13.701 (1) Å
α = 93.735 (1)°
β = 99.564 (1)°
γ = 102.363 (1)°
*V* = 546.03 (7) Å^3^
*Z* = 1
Mo *K*α radiation
μ = 3.75 mm^−1^
*T* = 200 K
0.34 × 0.26 × 0.17 mm

#### Data collection

Bruker SMART 1000 CCD diffractometer
Absorption correction: multi-scan (*SADABS*; Bruker, 2000 ▶) *T*_min_ = 0.797, *T*_max_ = 1.000
4051 measured reflections
2645 independent reflections
2040 reflections with *I* > 2σ(*I*)
*R*_int_ = 0.015

#### Refinement

*R*[*F* ^2^ > 2σ(*F* ^2^)] = 0.046
*wR*(*F* ^2^) = 0.152
*S* = 1.30
2645 reflections
145 parameters
H-atom parameters constrained
Δρ_max_ = 1.25 e Å^−3^
Δρ_min_ = −1.87 e Å^−3^

### 

Data collection: *SMART* (Bruker, 2000 ▶); cell refinement: *SAINT* (Bruker, 2000 ▶); data reduction: *SAINT*; program(s) used to solve structure: *SHELXS97* (Sheldrick, 2008 ▶); program(s) used to refine structure: *SHELXL97* (Sheldrick, 2008 ▶); molecular graphics: *ORTEP-3* (Farrugia, 1997 ▶) and *PLATON* (Spek, 2009 ▶); software used to prepare material for publication: *SHELXL97*.

## Supplementary Material

Crystal structure: contains datablocks global, I. DOI: 10.1107/S1600536810028813/ng5004sup1.cif

Structure factors: contains datablocks I. DOI: 10.1107/S1600536810028813/ng5004Isup2.hkl

Additional supplementary materials:  crystallographic information; 3D view; checkCIF report

## Figures and Tables

**Fig. 1.**
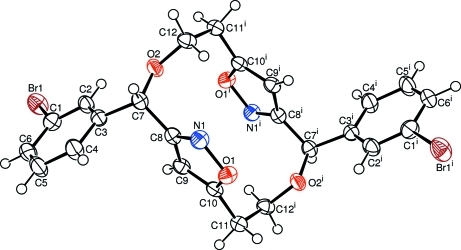
The structure of the title compound, with displacement ellipsoids drawn at the 50% probability level for non-H atoms [Symmetry code: (i) -x, -y, -z].

**Fig. 2.**
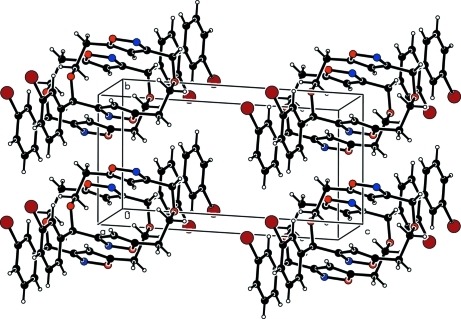
View of the unit-cell contents of the title compound.

## supplementary crystallographic information

### Comment

Many isoxazole derivatives are known to have a variety of biological activities in pharmaceutical and agricultural areas (Kim *et al.*, 1994, 1997; Lang & Lin, 1984). Recently we reported that the syntheses of various pyrano[3,4-*c*]isoxzole derivatives by means of the intramolecular 1,3-dipolar cycloaddition of a nitrile oxide containing an alkyne moiety within the structure and that these fused isoxazoles displayed fungicidal activities against some plant pathogens (Kim *et al.*, 1999). During the chromatographic purification of the crude product, we isolated an unexpected macrocylic isoxazole compound which was formed by intermolecular cycloaddition process.

The asymmetric unit of the title compound, C_24_H_20_Br_2_N_2_O_4_, contains one half of the formula unit; a centre of inversion is located at the midpoint of the compound (Fig. 1). The C7 and C11 atoms lie in the isoxazole ring plane with the largest deviation of 0.055 (9) Å (C7) from the least-squares plane of the isoxazole ring. The compound displays intra- and intermolecular π-π interactions between the isoxazole rings (the symmetry operations for second planes: -*x*,-*y*,-*z* and -*x*,1 - *y*,-*z*, respectively), the shortest centroid-centroid distance being 3.837 (3) Å and 3.634 (3) Å, respectively. The parallel planes are shifted for 1.048 Å and 1.936 Å, respectively (Fig. 2). There may also be weak intermolecular π-π interactions between adjacent benzene rings, with a shortest centroid-centroid distance of 4.453 (4) Å. The molecules are stacked in columns along the *a* axis and the Br···Br contacts are present. The shortest Br1···Br1*^a^* [symmetry code: (*a*) 2 - *x*,-*y*,1 - *z*] distance is 3.508 (1) Å.

### Experimental

A mixture of 1-bromo-3-[1-(but-3-ynyloxy)-2-nitroethyl]benzene (1.49 g, 5 mmol), phenyl isocyanate (2.97 g, 25 mmol) and Et_3_N (51 mg, 0.5 mmol) in dry benzene (30 ml) was stirred for 12 h at 25 °C under nitrogen atmosphere. Water (1 ml) was added and the mixture was stirred for 2 h at which time the solids were removed by vacuum filtration. The filtrate was dried (MgSO_4_) and concentrated *in vacuo* to give crude product, which was column chromatographed (SiO_2_) by eluting with a mixture of n-hexane/EtOAc (10:1) to afford the title compound (34 mg, 1.2%) as a white solid. Crystals suitable for X-ray analysis were obtained by slow evaporation from an n-hexane/EtOAc solution. Mp 231 °C. ^1^H NMR (600 MHz, CDCl_3_): δ 7.57 (s, 2H, Ar), 7.41–7.18 (m, 6H, Ar), 5.41 (s, 2H, isoxazole), 5.37 (s, 2H, –O—C*H*-C_6_H_4_Br), 4.19 (dt, 2H, J = 10.2 Hz, 3.0 Hz, –CH_2_C*H*H—O), 3.67 (bt, 2H, J = 10.2 Hz, –CH_2_CH*H*-O–), 3.15 (ddd, 2H, J = 16.2 Hz, 12.6 Hz, 3.0 Hz, –C*H*H—CH_2_O–), 2.77 (bd, J = 16.2 Hz, –CH*H*-CH_2_O–). ^13^C NMR (150 MHz, CDCl_3_): δ 172.71, 164.13, 141.11, 131.00, 129.98, 128.74, 124.43, 122.61, 98.90, 74.22, 67.40, 27.91.

### Refinement

H atoms were positioned geometrically and allowed to ride on their respective parent atoms [C—H = 0.95 (CH, *sp*^2^), 1.00 (CH, *sp*^3^) or 0.99 Å (CH_2_) and *U*_iso_(H) = 1.2*U*_eq_(C)]. The highest peak (1.25 e Å^-3^) and the deepest hole (-1.87 e Å^-3^) in the difference Fourier map are located 1.46 Å and 0.89 Å from the Br1 atom, respectively.

### Crystal data

**Table:**

C_24_H_20_Br_2_N_2_O_4_	*Z* = 1
*M**_r_* = 560.24	*F*(000) = 280
Triclinic, *P*1	*D*_x_ = 1.704 Mg m^−^^3^
Hall symbol: -P 1	Mo *K*α radiation, λ = 0.71073 Å
*a* = 5.6446 (4) Å	Cell parameters from 2388 reflections
*b* = 7.3703 (5) Å	θ = 2.8–28.1°
*c* = 13.701 (1) Å	µ = 3.75 mm^−^^1^
α = 93.735 (1)°	*T* = 200 K
β = 99.564 (1)°	Plate, colorless
γ = 102.363 (1)°	0.34 × 0.26 × 0.17 mm
*V* = 546.03 (7) Å^3^	

### Data collection

**Table:**

Bruker SMART 1000 CCD diffractometer	2645 independent reflections
Radiation source: fine-focus sealed tube	2040 reflections with *I* > 2σ(*I*)
graphite	*R*_int_ = 0.015
φ and ω scans	θ_max_ = 28.3°, θ_min_ = 2.8°
Absorption correction: multi-scan (*SADABS*; Bruker, 2000)	*h* = −7→6
*T*_min_ = 0.797, *T*_max_ = 1.000	*k* = −9→9
4051 measured reflections	*l* = −16→18

### Refinement

**Table:**

Refinement on *F*^2^	Primary atom site location: structure-invariant direct methods
Least-squares matrix: full	Secondary atom site location: difference Fourier map
*R*[*F*^2^ > 2σ(*F*^2^)] = 0.046	Hydrogen site location: inferred from neighbouring sites
*wR*(*F*^2^) = 0.152	H-atom parameters constrained
*S* = 1.30	*w* = 1/[σ^2^(*F*_o_^2^) + (0.*P*)^2^ + 3.5324*P*] where *P* = (*F*_o_^2^ + 2*F*_c_^2^)/3
2645 reflections	(Δ/σ)_max_ < 0.001
145 parameters	Δρ_max_ = 1.25 e Å^−^^3^
0 restraints	Δρ_min_ = −1.87 e Å^−^^3^

### Special details

**Table:**

Geometry. All e.s.d.'s (except the e.s.d. in the dihedral angle between two l.s. planes) are estimated using the full covariance matrix. The cell e.s.d.'s are taken into account individually in the estimation of e.s.d.'s in distances, angles and torsion angles; correlations between e.s.d.'s in cell parameters are only used when they are defined by crystal symmetry. An approximate (isotropic) treatment of cell e.s.d.'s is used for estimating e.s.d.'s involving l.s. planes.
Refinement. Refinement of *F*^2^ against ALL reflections. The weighted *R*-factor *wR* and goodness of fit *S* are based on *F*^2^, conventional *R*-factors *R* are based on *F*, with *F* set to zero for negative *F*^2^. The threshold expression of *F*^2^ > σ(*F*^2^) is used only for calculating *R*-factors(gt) *etc*. and is not relevant to the choice of reflections for refinement. *R*-factors based on *F*^2^ are statistically about twice as large as those based on *F*, and *R*- factors based on ALL data will be even larger.

### Fractional atomic coordinates and isotropic or equivalent isotropic displacement parameters (Å^2^)

**Table:**

	*x*	*y*	*z*	*U*_iso_*/*U*_eq_	
Br1	0.78777 (12)	0.13416 (10)	0.45968 (6)	0.0464 (2)	
O1	−0.0873 (7)	0.2957 (6)	−0.0511 (3)	0.0304 (9)	
O2	−0.0557 (7)	−0.0026 (5)	0.2100 (3)	0.0323 (9)	
N1	−0.1737 (8)	0.2688 (7)	0.0394 (4)	0.0315 (10)	
C1	0.5786 (11)	0.2773 (8)	0.3908 (4)	0.0336 (12)	
C2	0.3670 (11)	0.1843 (8)	0.3295 (4)	0.0333 (12)	
H2	0.3226	0.0517	0.3216	0.040*	
C3	0.2166 (10)	0.2874 (8)	0.2784 (4)	0.0288 (11)	
C4	0.2855 (12)	0.4808 (8)	0.2927 (5)	0.0383 (14)	
H4	0.1827	0.5521	0.2588	0.046*	
C5	0.5037 (13)	0.5714 (9)	0.3561 (5)	0.0443 (16)	
H5	0.5493	0.7040	0.3655	0.053*	
C6	0.6540 (12)	0.4683 (9)	0.4054 (5)	0.0411 (15)	
H6	0.8049	0.5278	0.4482	0.049*	
C7	−0.0170 (10)	0.1934 (7)	0.2048 (4)	0.0286 (11)	
H7	−0.1606	0.2385	0.2229	0.034*	
C8	0.0115 (9)	0.2363 (7)	0.1015 (4)	0.0254 (11)	
C9	0.2206 (10)	0.2402 (7)	0.0570 (4)	0.0273 (11)	
H9	0.3764	0.2208	0.0867	0.033*	
C10	0.1503 (9)	0.2772 (7)	−0.0368 (4)	0.0263 (11)	
C11	0.2785 (10)	0.2954 (8)	−0.1230 (4)	0.0303 (12)	
H11A	0.4454	0.3774	−0.1016	0.036*	
H11B	0.1857	0.3539	−0.1749	0.036*	
C12	−0.2998 (10)	−0.1054 (8)	0.1658 (4)	0.0310 (12)	
H12A	−0.3729	−0.0385	0.1125	0.037*	
H12B	−0.4066	−0.1201	0.2166	0.037*	

### Atomic displacement parameters (Å^2^)

**Table:**

	*U*^11^	*U*^22^	*U*^33^	*U*^12^	*U*^13^	*U*^23^
Br1	0.0331 (3)	0.0454 (4)	0.0559 (4)	0.0092 (3)	−0.0055 (3)	0.0045 (3)
O1	0.0248 (19)	0.038 (2)	0.031 (2)	0.0108 (16)	0.0029 (16)	0.0109 (17)
O2	0.029 (2)	0.029 (2)	0.032 (2)	−0.0001 (16)	−0.0033 (16)	0.0014 (16)
N1	0.023 (2)	0.040 (3)	0.032 (3)	0.008 (2)	0.0034 (19)	0.005 (2)
C1	0.034 (3)	0.034 (3)	0.033 (3)	0.011 (2)	0.005 (2)	0.006 (2)
C2	0.035 (3)	0.027 (3)	0.035 (3)	0.002 (2)	0.009 (2)	−0.006 (2)
C3	0.031 (3)	0.026 (3)	0.027 (3)	0.004 (2)	0.003 (2)	0.003 (2)
C4	0.042 (3)	0.029 (3)	0.038 (3)	0.007 (3)	−0.006 (3)	0.002 (3)
C5	0.052 (4)	0.026 (3)	0.042 (4)	−0.004 (3)	−0.008 (3)	0.000 (3)
C6	0.033 (3)	0.044 (4)	0.035 (3)	−0.008 (3)	−0.004 (3)	0.002 (3)
C7	0.028 (3)	0.025 (3)	0.031 (3)	0.006 (2)	0.003 (2)	0.000 (2)
C8	0.025 (3)	0.022 (2)	0.030 (3)	0.007 (2)	0.002 (2)	0.002 (2)
C9	0.022 (2)	0.026 (3)	0.034 (3)	0.005 (2)	0.002 (2)	0.004 (2)
C10	0.022 (2)	0.022 (2)	0.032 (3)	0.0018 (19)	0.002 (2)	0.004 (2)
C11	0.029 (3)	0.027 (3)	0.032 (3)	0.002 (2)	0.005 (2)	0.004 (2)
C12	0.023 (3)	0.031 (3)	0.038 (3)	0.003 (2)	0.005 (2)	0.007 (2)

### Geometric parameters (Å, °)

**Table:**

Br1—C1	1.921 (6)	C5—H5	0.9500
O1—C10	1.360 (6)	C6—H6	0.9500
O1—N1	1.416 (6)	C7—C8	1.497 (8)
O2—C7	1.423 (6)	C7—H7	1.0000
O2—C12	1.433 (6)	C8—C9	1.412 (7)
N1—C8	1.308 (7)	C9—C10	1.344 (7)
C1—C2	1.358 (8)	C9—H9	0.9500
C1—C6	1.372 (9)	C10—C11	1.483 (8)
C2—C3	1.391 (8)	C11—C12^i^	1.520 (8)
C2—H2	0.9500	C11—H11A	0.9900
C3—C4	1.386 (8)	C11—H11B	0.9900
C3—C7	1.521 (7)	C12—C11^i^	1.520 (8)
C4—C5	1.391 (8)	C12—H12A	0.9900
C4—H4	0.9500	C12—H12B	0.9900
C5—C6	1.381 (9)		
			
C10—O1—N1	107.8 (4)	O2—C7—H7	109.7
C7—O2—C12	114.0 (4)	C8—C7—H7	109.7
C8—N1—O1	105.5 (4)	C3—C7—H7	109.7
C2—C1—C6	123.7 (6)	N1—C8—C9	111.9 (5)
C2—C1—Br1	118.4 (5)	N1—C8—C7	120.4 (5)
C6—C1—Br1	117.9 (5)	C9—C8—C7	127.6 (5)
C1—C2—C3	118.6 (5)	C10—C9—C8	104.7 (5)
C1—C2—H2	120.7	C10—C9—H9	127.6
C3—C2—H2	120.7	C8—C9—H9	127.6
C4—C3—C2	119.1 (5)	C9—C10—O1	110.0 (5)
C4—C3—C7	119.1 (5)	C9—C10—C11	132.4 (5)
C2—C3—C7	121.7 (5)	O1—C10—C11	117.5 (5)
C3—C4—C5	120.7 (6)	C10—C11—C12^i^	110.7 (5)
C3—C4—H4	119.6	C10—C11—H11A	109.5
C5—C4—H4	119.6	C12^i^—C11—H11A	109.5
C6—C5—C4	119.8 (6)	C10—C11—H11B	109.5
C6—C5—H5	120.1	C12^i^—C11—H11B	109.5
C4—C5—H5	120.1	H11A—C11—H11B	108.1
C1—C6—C5	118.0 (6)	O2—C12—C11^i^	107.4 (4)
C1—C6—H6	121.0	O2—C12—H12A	110.2
C5—C6—H6	121.0	C11^i^—C12—H12A	110.2
O2—C7—C8	109.7 (4)	O2—C12—H12B	110.2
O2—C7—C3	107.8 (4)	C11^i^—C12—H12B	110.2
C8—C7—C3	110.1 (5)	H12A—C12—H12B	108.5
			
C10—O1—N1—C8	0.0 (6)	C2—C3—C7—C8	−113.0 (6)
C6—C1—C2—C3	−0.1 (10)	O1—N1—C8—C9	0.0 (6)
Br1—C1—C2—C3	−179.2 (4)	O1—N1—C8—C7	−177.6 (4)
C1—C2—C3—C4	−0.8 (9)	O2—C7—C8—N1	100.4 (6)
C1—C2—C3—C7	177.1 (6)	C3—C7—C8—N1	−141.0 (5)
C2—C3—C4—C5	0.8 (10)	O2—C7—C8—C9	−76.8 (7)
C7—C3—C4—C5	−177.1 (6)	C3—C7—C8—C9	41.8 (7)
C3—C4—C5—C6	0.1 (11)	N1—C8—C9—C10	0.0 (6)
C2—C1—C6—C5	1.1 (10)	C7—C8—C9—C10	177.4 (5)
Br1—C1—C6—C5	−179.9 (5)	C8—C9—C10—O1	0.0 (6)
C4—C5—C6—C1	−1.0 (11)	C8—C9—C10—C11	−178.2 (5)
C12—O2—C7—C8	−76.1 (6)	N1—O1—C10—C9	0.0 (6)
C12—O2—C7—C3	164.0 (5)	N1—O1—C10—C11	178.6 (4)
C4—C3—C7—O2	−175.5 (5)	C9—C10—C11—C12^i^	73.1 (7)
C2—C3—C7—O2	6.6 (7)	O1—C10—C11—C12^i^	−105.0 (5)
C4—C3—C7—C8	64.8 (7)	C7—O2—C12—C11^i^	147.7 (5)

Symmetry codes: (i) −*x*, −*y*, −*z*.
